# Are Ponto‐Caspian species able to cross salinity barriers? A case study of the gammarid *Pontogammarus maeoticus*

**DOI:** 10.1002/ece3.4461

**Published:** 2018-09-05

**Authors:** Nora‐Charlotte Pauli, Filipa Paiva, Elizabeta Briski

**Affiliations:** ^1^ GEOMAR Helmholtz Centre for Ocean Research Kiel Kiel Germany; ^2^ Christian‐Albrechts Universität Kiel Kiel Germany; ^3^ MARE – Marine and Environmental Sciences Centre Quinta do Lorde Marina Caniçal, Madeira Island Portugal; ^4^ Present address: Institute for Chemistry and Biology of the Marine Environment Carl‐von‐Ossietzky University Oldenburg Germany

**Keywords:** freshwater conditions, freshwater origin, heritability, marine conditions, selection experiment, standing genetic variation

## Abstract

Recently, Ponto‐Caspian species (i.e., area of Azov, Black, and Caspian Seas) have invaded brackish and freshwater habitats of the North and Baltic Seas and the Laurentian Great Lakes in much higher numbers than expected based on shipping frequency and environmental conditions among these regions. Therefore, it has been suggested that Ponto‐Caspian species may have inherent advantages over other species in colonizing new habitats, or that they are of freshwater origin. Artificial selection offers the possibility to investigate phenotypic plasticity, shifts in environmental tolerance, and heritability of environmentally sensitive traits; therefore, in this study, we conducted artificial selection experiments on Ponto‐Caspian amphipod *Pontogammarus maeoticus* collected from 10 PSU to evaluate adaptation capacity of this species to different salinities, and to shed additional light on a possible freshwater origin of Ponto‐Caspian invaders. Our results indicated that selection to lower salinity than that of the population's ambient salinity is possible within few generations due to a likely existence of standing polymorphic variation for selection to act on. In contrast, selection to higher salinity was unsuccessful because the phenotypic variation was mainly caused by environmental variance and therefore might depend on new mutations. Consequently, the results of our study suggest that the tested species might be of freshwater origin and lacks necessary genetic background for adaptation to fully marine conditions. Further selection studies using more species and populations, as well as molecular techniques, should be conducted to elucidate if other Ponto‐Caspian invaders are of freshwater origin as well.

## 1 INTRODUCTION

Human‐mediated spread of species has been accelerating in the past decades (Hulme, 2009), with commercial shipping being the most important introduction vector in aquatic habitats (Carlton & Geller, 1993). Biological invasion is a multistage process that includes transport, introduction, establishment, and spread (Blackburn et al., 2011; Lockwood, Hoopes, & Marchetti, 2013). Several factors affect invasion success including propagule pressure (i.e., introduction effort, number of introduced individuals), environmental conditions during transport and in recipient habitats, and integration of introduced populations to recipient communities (Blackburn et al., 2011; Simberloff, 2009). As transport and novel environments may pose strong selection pressure on an introduced population (Briski et al., 2018; Lee, Remfert, & Gelembiuk, 2003), standing genetic variation of the population seems to be more important than new mutations (Bock et al., 2015; Dlugosch, Anderson, Braasch, Cang, & Gillette, 2015). Therefore, establishment success depends on phenotypic plasticity and adaptation capacity of introduced populations to different environmental factors (Blackburn et al., 2011; Hoffmann & Hercus, 2000).

Salinity is one of the most important factors influencing spread and establishment of aquatic species, as organisms need to maintain homeostasis balancing the ion concentration of their inner body fluids in relation to the ion concentration of the surrounding water to avoid (de‐) hydration (Sutcliffe, 1978). However, the establishment of marine and brackish species in freshwater habitats has been reported in recent years, with many of those species originating from the Ponto‐Caspian region (i.e., Azov, Black, and Caspian Seas; Casties, Seebens, & Briski, 2016; Lee & Bell, 1999; Ricciardi & Macisaac, 2000; Ruiz, Carlton, Grosholz, & Hines, 1997), while *vice versa* invasions rarely occur (Ricciardi & Macisaac, 2000). Furthermore, recent studies demonstrated that Ponto‐Caspian species invaded brackish waters of the Baltic Sea and freshwater of the Laurentian Great Lakes in much higher numbers than was expected based on shipping frequency and environmental conditions among native and invaded regions (Casties et al., 2016). In addition, studies determining the distribution of several Ponto‐Caspian gammarids and their salinity preference in invaded areas of Northern Europe demonstrated mixed results among species and freshwater and brackish habitats (Kobak et al., 2017; Dobrzycka‐Krahel & Graca, 2018), while comparative assessment of salinity tolerance of six populations of two Ponto‐Caspian gammarids revealed higher tolerance to freshwater compared to fully marine conditions (Paiva et al., 2018). Moreover, molecular analysis of mitochondrial DNA revealed a marked genetic divergence between Caspian and Black Sea populations of *P. maeoticus*, as well as of other Ponto‐Caspian crustaceans (Cristescu, Hebert, & Onciu, 2003). Consequently, if Ponto‐Caspian taxa adapt easily to different salinities, in particular to freshwater conditions, current management strategies to prevent new invasions should consider this adaptation capacity in the future.

A major part of Ponto‐Caspian fauna is predominately represented by crustaceans (Cristescu & Hebert, 2005; Mordukhai‐Boltovskoi, 1964), which also constitute a large proportion of nonindigenous species in European and North American freshwaters (Mills, Leach, Carlton, & Secor, 1993; Streftaris, Zenetos, & Papathanassiou, 2005). Within Crustacea, the family Gammaridae (order Amphipoda) is one of the most successful groups invaders from this region, which includes high impact species such as *Dikerogammarus villosus*,* D*. *haemobaphes*, and *Echinogammarus ischnus* (Cristescu, Witt, Grigorovich, Hebert, & Macisaac, 2004; Dick, Platvoet, & Kelly, 2002). For example, *D*. *villosus* and *Pontogammarus robustoides* were associated with a severe decline in abundance of native amphipods by competitive exclusion and intraguild predation in European rivers and lakes (Arbačiauskas & Gumuliauskaitė, 2007; Dick et al., 2002). Similar to crustaceans, other Ponto‐Caspian taxa had also major ecological impacts on invaded ecosystems, such as the zebra mussel *Dreissena polymorpha,* which is responsible for severe alterations in water transparency, nutrient cycling and decreasing biomass of unionid bivalves, as well as the round goby *Neogobius melanostomus* that competitively excluded native species due to diet and habitat overlap in the Great Lakes, brackish waters of the Baltic Sea, and European freshwaters (Kornis, Mercado‐Silva, & Vander Zanden, 2012; Ojaveer, Leppäkoski, Olenin, & Ricciardi, 2002; Dzierżyńska‐Białończyk, Jermacz, Maćkiewicz, Gajewska, & Kobak, 2018).

As artificial selection offers the possibility to investigate phenotypic plasticity, shifts in environmental tolerance, and heritability of environmentally sensitive traits, in this study, we conducted artificial selection experiments on Ponto‐Caspian amphipod *Pontogammarus maeoticus* (Sovinskij, 1894) collected from 10 PSU to evaluate the adaptation capacity of this species to different salinities, and to assess a possible freshwater origin of Ponto‐Caspian invaders. *P*. *maeoticus* has been chosen due to its euryhalinity, invasion record of inland waterbodies and reservoirs of Ukraine and Turkey (Alexandrov et al., 2007; Kocataş, Katagan, Özbek, & Sezgin, 2003; Özbek, 2011), wide spread distribution and high abundance in its native region (Mirzajani, 2003; Mordukhai‐Boltovskoi, 1964), and consecutive breeding and short generation time (1–2 months; Nazarhaghighi, Shabanipour, Zarghami, & Etemadi‐Deylami, 2013). During the experiments, we followed mortality of adults, hatching success, and growth and survival of offspring in different salinities. We tested three hypotheses: (a) selection is possible to both directions, to higher and lower salinity; (b) the population selected for low salinity will perform better in lower salinity; and (c) the population selected for high salinity will perform better in higher salinity.

## 2 METHODS

### 2.1 Specimen collection

**Figure 1:**
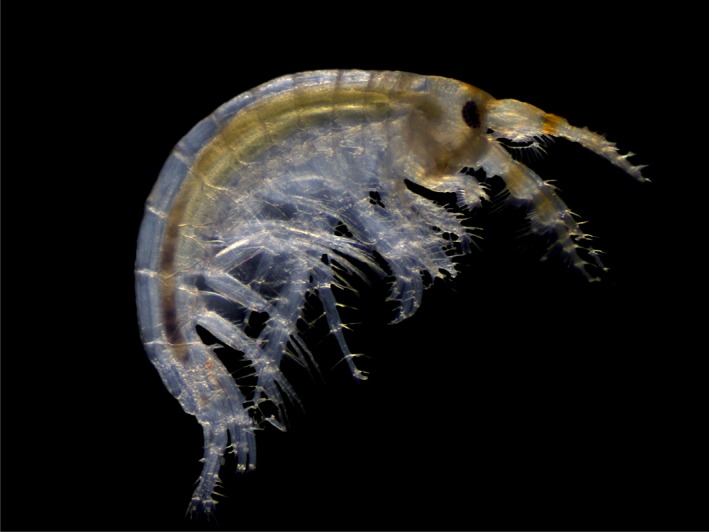
Image of a juvenile specimen (1 month old) of the tested species *Pontogammarus maeoticus* (Sovinskij, 1894). Photo credit Nora‐Charlotte Pauli

Specimens of *Pontogammarus maeoticus* (Figure 1) were collected in October 2014 in the Southern Caspian Sea near Jafrud, Iran (37°29′09″N, 49°30′20″E). Salinity and temperature of the sampling site were 10.6 PSU and 18°C, respectively. After sampling, animals were identified morphologically (Moiceiev & Filatova, 1985; Sars, 1896; Stock, 1974; Stock, Mirzajani, Vonk, Naderi, & Kiabi, 1998), and 96 individuals were transported to Kiel, Germany. Prior to the experiments, animals were reared in the laboratory under similar conditions to their native habitat: the same salinity, temperature, and medium grain‐sized sediment. Density of the laboratory population was between 50 and 100 individuals per 56 L tank.

### 2.2 Selection to different salinities

The selection to different salinities started in April 2017 and consisted of two treatments and a control: “selected 4 PSU,” “selected 16 PSU,” and “ambient 10 PSU,” hereafter referred to as selection treatments. From the laboratory population reared at 10 PSU, 15–20 adults and 20–40 juveniles were transferred without acclimatization to each of the thirteen 56 L tanks (i.e., five tanks of 4 PSU, five tanks of 16 PSU, and three tanks of 10 PSU, respectively). Selection was conducted for 15 weeks corresponding to approximately 1–2 generations (Nazarhaghighi et al., 2013). Animals were reared under the above‐mentioned laboratory conditions, except that salinity was varied, and fed ad libitum with a mixture of fish food flakes, pellets, and green algae.

### 2.3 Salinity stress experiment of selected populations

To assess fitness of the selected populations, a salinity stress experiment was conducted to test survival of adults, hatching success, and growth and survival of juveniles in different salinities. The experiment consisted of three conditions: (a) control (corresponding to the respective selection treatment salinity, i.e., “selected 4 PSU,” “selected 16 PSU,” and “ambient 10 PSU”); (b) decreasing salinity; and (c) increasing salinity, hereafter referred to as “control,” “low stress,” and “high stress” experimental condition, respectively. Each experimental condition was conducted in four replicates. Each replicate started with five randomly chosen couples, or when there were not enough couples available randomly chosen single adult animals to reach ten animals per tank. The sex ration was equal in all experimental tanks.

The salinity of the “control” experimental condition was identical to the selection treatment salinities (i.e., 4 PSU, 10 PSU, and 16 PSU). The “low stress” and “high stress” experimental condition began at the respective selection treatment salinity of the population, which was then increased/decreased by 2 PSU every 2 days, respectively, until reaching 40 PSU and 0 PSU (Delgado, Guerao, & Ribera, 2011; Normant, Feike, Szaniawska, & Graf, 2007). Increased and decreased salinities were achieved by adding artificial seawater (Instant Ocean^®^) or potable tap water to filtered Kiel Fjord water (20 μm). Water was exchanged by removing half of the water from the tank and replacing it with new water of the salinity necessary to achieve the desired salinity. To apply the same disturbance to the “control” experimental condition, water was also exchanged every 2 days. Survival of adults was documented daily until 2 weeks after reaching 40 PSU and 0 PSU. Every second day, before water exchange, tanks were searched for newly hatched juveniles, which were then separated from the parents and reared at approximate hatching salinity for 6 weeks. Juveniles from two hatches, corresponding to two salinity steps, were reared together at the intermediate salinity of the two salinity steps. Hatching success was assessed as the total number of juveniles per hatch, including dead individuals found in the tanks. Juvenile survival was recorded every second week, as well as juvenile growth which was measured using the cephalon length as a proxy for total length (Delgado et al., 2011; Lancellotti & Trucco, 1993,). Cephalon length was measured by a stereomicroscope (Stemi 508, ZEISS) using a 20‐fold magnification and the ZEN software (*vs*. 2.3, ZEISS). The primary datasets containing experimental results are available at PANGAEA, https://doi.org/10.1594/pangaea.887340.

### 2.4 Heritability estimation

As heritability is expressed as the ratio of phenotypic variability caused by genetic variance, it partly determines how fast the mean phenotype evolves in response to selection (Conner & Hartl, 2004). Selection changes the array of gene frequencies leading to observable changes in the population mean (Falconer & Mackay, 1996), therefore allowing heritability estimates by comparing population means of the parental and selected offspring populations (Briski, Van Stappen, Bossier, & Sorgeloos, 2008; Hetzel, Crocos, Davis, Moore, & Preston, 2000). Thus, here we estimated heritability of juvenile survival and growth from offspring–midparent regression (Conner & Hartl, 2004; Falconer & Mackay, 1996). For this, the median number of offspring per hatching salinity and the median cephalon length per family (replicate tank) and cohort (hatching salinity) were regressed on the median number of offspring in ambient salinity and midparent median cephalon length (both parents), respectively. The median was used for both variables as data were nonnormally distributed. Linear regression was fitted following the equation (Conner & Hartl, 2004):

(1) y=a+bx

with the slope *b* used as heritability estimate for offspring–midparent regression (Falconer & Mackay, 1996):

(2) $$b=h^{2}$$

The unselected control population (i.e., “ambient 10 PSU”) was considered as parental generation and was compared to the “selected 4 PSU” and “selected 16 PSU” populations, respectively.

### 2.5 Data analyses

The effect of the independent variables—selection treatment (i.e., “selected 4 PSU,” “selected 16 PSU,” and “ambient 10 PSU”) and experimental condition (i.e., “control,” “low stress,” and “high stress”)—on the dependent variables—adult survival, juvenile survival, and cephalon length—as well as their interactions was tested using the software R, version 3.3.3 (R Core Team, 2017). The effect of selection treatment and experimental condition on adult survival was tested using the Scheirer–Ray–Hare test in the rcompanion package for R (Mangiafico, 2017) after testing for the assumption of normality and homogeneity of variances. Multiple comparisons were conducted by Dunn's test with Bonferroni adjustment using the eponymous package in R (Dinno, 2017). The effect of selection treatment and experimental condition on juvenile cephalon length was tested by a generalized linear model (glm) on the log transformed data after checking for the validity of assumptions. The effect on juvenile survival was tested by a glm of the gamma family. Pairwise contrasts for all possible treatment combinations of both dependent variables (i.e., juvenile growth and survival) were calculated using the lsmeans function of the eponymous package in R applying a Bonferroni adjustment (Lenth, 2016). Data visualization of dependent variables was conducted by the ggplot2 package (Wickham & Chang, 2016).

## 3 RESULTS

### 3.1 Survival of adults

**Figure 2:**
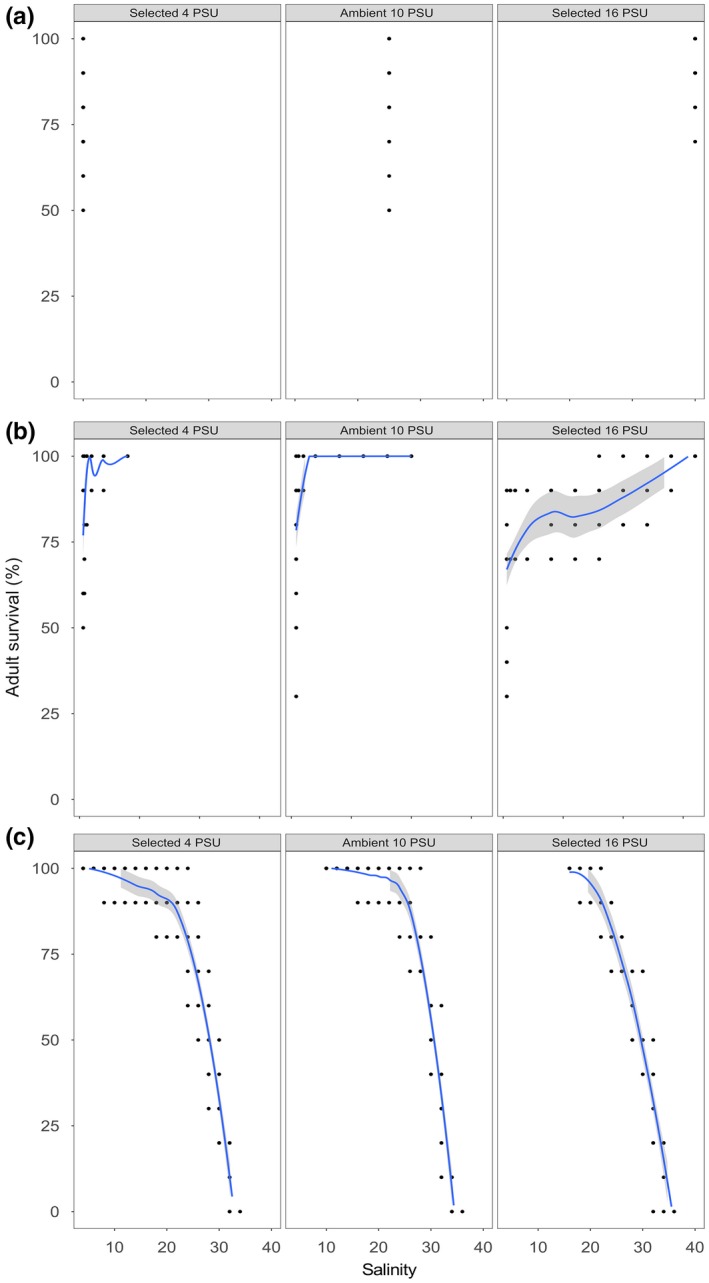
Survival of adults in “control” (a), “low stress” (b), and “high stress” (*c*) experimental conditions. Columns represent selection treatments (i.e., “selected 4 PSU,” “ambient 10 PSU,” and “selected 16 PSU,” respectively). Curves and respective confidence intervals (95%; gray area) were fitted using the method “loess” in R

Mean survival of adults in “control” experimental condition ranged between 72.5% (“selected 4 PSU”) and 85% (“selected 16 PSU”; Figure 2). Selection treatment, experimental condition, and their interaction significantly affected adult survival (*p* < 0.001, *p* = 0.004, *p* < 0.001, respectively; Table 1). Survival in “low stress” experimental condition was highest for the “selected 4 PSU” treatment (75%) and lowest for the “selected 16 PSU” treatment (52.5%; Figure 2). In “high stress” experimental condition, no animals survived salinity above 34 PSU, irrespective of the selection treatment (Figure 2); however, the onset of mortality was earlier for the “selected 4 PSU” treatment compared to “selected 16 PSU” and “ambient 10 PSU” treatments. Overall, survival of all selection treatments was significantly lower in “high stress” compared to “low stress” and “control” experimental conditions (*p* = 0.014; Supporting information Table S1). Survival for both “selected 4 PSU” and “selected 16 PSU” treatments differed significantly from “ambient 10 PSU” in all experimental conditions (*p* < 0.001; Supporting information Table S1).

**Table 1:** Results of the Scheirer–Ray–Hare test of the effect of selection treatment, experimental condition and their interaction on adult survival

Adult survival	Df	Sum Sq	H	*p*‐value
Selection treatment	2	1251420	21.828	<0.001
Experimental condition	2	642194	11.201	0.004
Selection treatment: Experimental condition	4	5478465	95.558	<0.001
Residuals	861	42448709		<0.001

Df, Sum Sq, and H denote degrees of freedom, sum of squares, and the test statistic, respectively.

### 3.2 Survival and hatching success of juveniles

**Figure 3:**
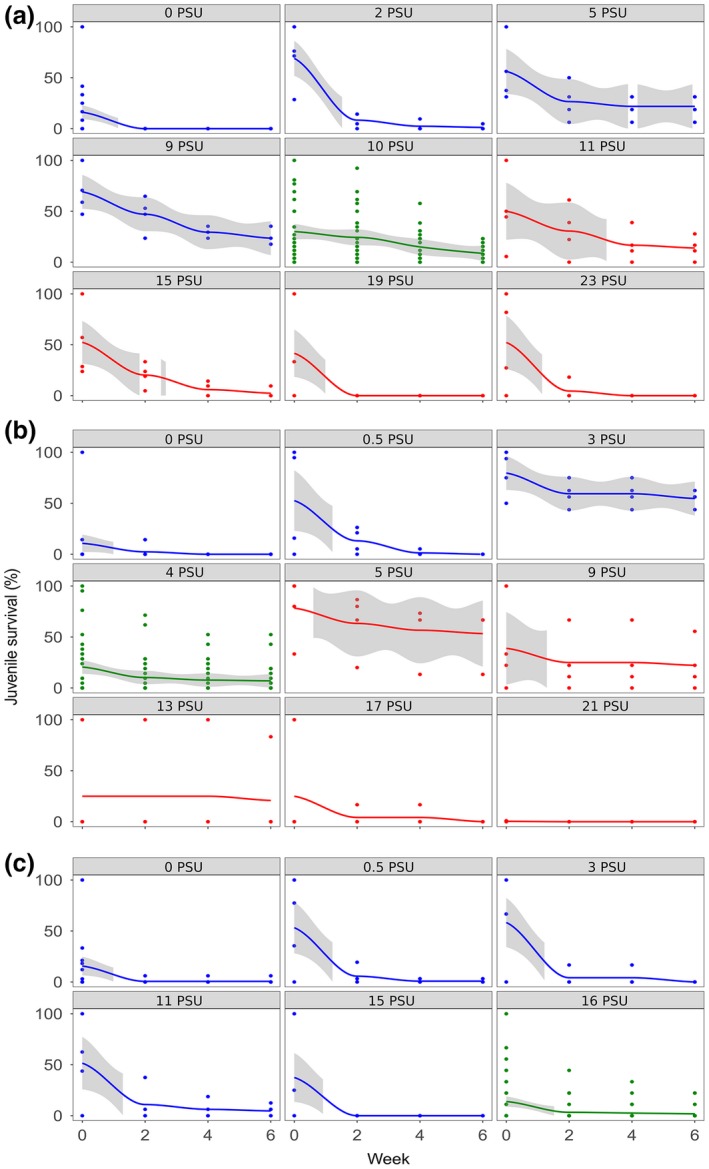
Survival of juveniles in “ambient 10 PSU” (a), “selected 4 PSU” (b), and “selected 16 PSU” (c) treatments over time. Columns represent hatching salinities of the respective juvenile cohorts (i.e., hatching salinity). Experimental conditions are depicted in: blue (“low stress”), green (“control”), and red (“high stress”). Curves and respective confidence intervals (95%; gray area) were fitted using the method “loess” in R

Juveniles hatched in salinities from 0 PSU to 23 PSU (Figure 3). No successful reproduction was documented for the “selected 16 PSU” treatment in “high stress” experimental condition. However, successful reproduction was recorded for all selection treatments in freshwater (<0.5 PSU), which was reflected in the significant effect of “low stress” experimental condition compared to the other two experimental conditions (*p* = 0.035; Table 2). The maximum number of juveniles hatched per salinity and replicate was 33 (i.e., “selected 16 PSU” in “low stress”) with an overall mean of 2.1 individuals per salinity step and replicate. Juvenile survival decreased over the period of 6 weeks in all selection treatments and experimental combinations. The highest survival after 6 weeks was observed for the “selected 4 PSU” treatment with 54.68 % of individuals survived at 3 PSU and 53.33 % at 5 PSU (Figure 3). In the “ambient 10 PSU” treatment, survival was highest at 9 PSU and 5 PSU with 23.53 % and 21.88 % of individuals survived, respectively (Figure 3). The “selected 16 PSU” treatment demonstrated the highest survival at 11 PSU (4.69%; Figure 3). There was a significant effect on juvenile survival for the “selected 16 PSU” treatment, with a significantly higher survival in “low stress” compared to “control” experimental condition (*p* = 0.014; Table 2), while there was no significant difference between “control” and “high stress,” nor between “low stress” and “high stress” experimental conditions.

**Table 2:** Results of the generalized linear model analysis of the effect of selection treatments, experimental condition and their interaction on juvenile survival using the gamma family

Juvenile survival	Estimate	Std. Error	*T*‐value	*p*‐value
Intercept	0.189	0.022064	8.591	**<0.001**
“Ambient 10 PSU” treatment	−0.026	0.026694	−0.978	0.329
“Selected 16 PSU” treatment	0.171	0.071648	2.388	**0.018**
“High stress” experimental condition	−0.025	0.032576	−0.77	0.442
“Low stress” experimental condition	−0.065	0.03042	−2.122	**0.035**
“Ambient 10 PSU” treatment: “High stress” exp. condition	0.036	0.045342	0.79	0.430
“Selected 16 PSU” treatment: “High stress” exp. condition	−0.002	0.305009	−0.007	0.994
“Ambient 10 PSU” treatment: “Low stress” exp. condition	0.064	0.039807	1.603	0.109
“Selected 16 PSU” treatment: “Low stress” exp. condition	−0.141	0.078415	−1.791	0.074

*T*‐value denotes the test statistic.

Significant values are highlighted in bold

### 3.3 Cephalon length

**Figure 4:**
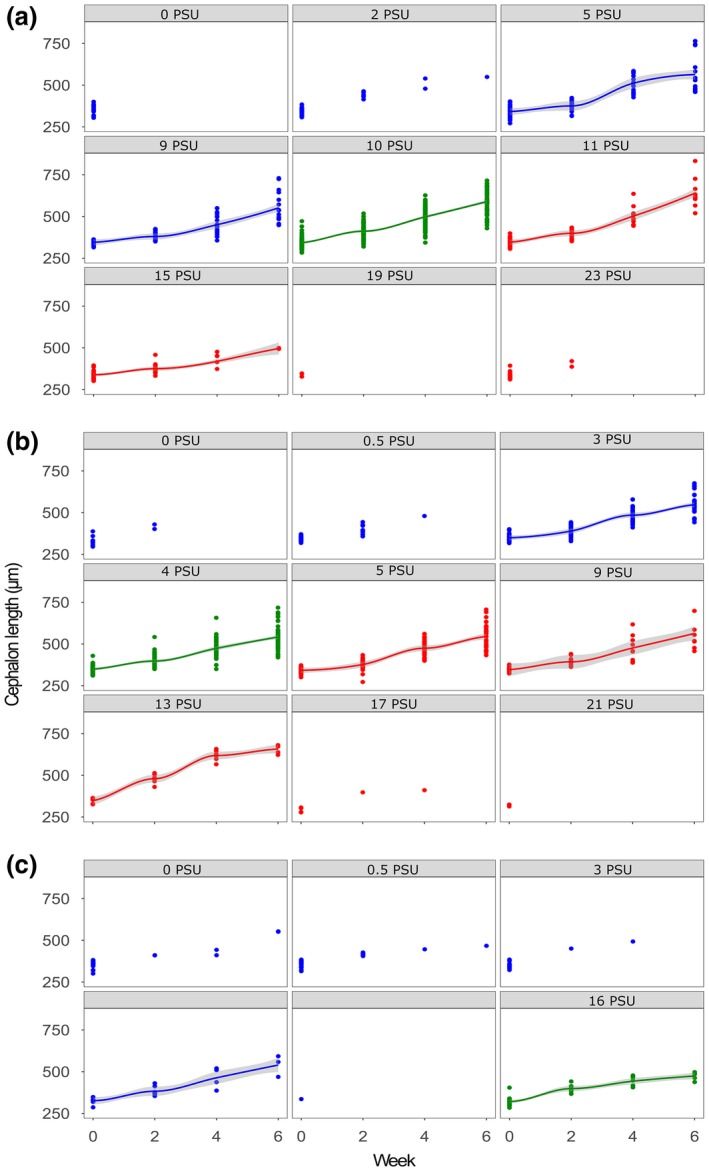
Cephalon length of juveniles [μm] in “ambient 10 PSU” (a), “selected 4 PSU” (b), and “selected 16 PSU” (c) treatments over time. Columns represent hatching salinities of the respective juvenile cohorts (i.e., hatching salinity). Experimental conditions are depicted in: blue (“low stress”), green (“control”), and red (“high stress”). Curves and respective confidence intervals (95%; gray area) were fitted using the method “loess” in R

Mean cephalon length increased over the period of 6 weeks in all selection treatment and experimental condition combinations. Minimum length was 271.8 μm (in week 0) and the maximum was 832.7 μm (in week 6 for “ambient 10 PSU” at 11 PSU; overall median = 382.8 μm). Cephalon length of the “ambient 10 PSU” treatment differed significantly between “control” and each of the experimental conditions (i.e., “low stress” and “high stress,” Figure 4) with the smallest cephalon in “high stress” and largest in “control” experimental condition, which was reflected by the significant interaction of “ambient 10 PSU” treatment with “high stress” and “low stress” experimental conditions (*p* < 0.001 and *p* = 0.006, respectively; Table 3), as well as by pairwise comparisons of the respective treatment combinations (control—high stress, *p* < 0.001; control—low stress, *p* > 0.001; Supporting information Table S3). Cephalon length of the “selected 16 PSU” treatment differed significantly from all other selection treatments (*p* < 0.001; Table 3). The juveniles from the “selected 16 PSU” treatment had a significantly shorter cephalon length than juveniles from “selected 4 PSU,” and “ambient 10 PSU” treatments under control experimental condition (both *p* < 0.001; Supporting information Table S3). For the “selected 4 PSU” treatment, cephalon length did not differ significantly among experimental conditions (Figure 4). Overall, “high stress” experimental condition had a significant effect on cephalon length (*p* = 0.016; Table 3); juveniles from “ambient 10 PSU” had significantly larger cephalon than juveniles from “selected 4 PSU” treatment (*p* < 0.001; Supporting information Table S3).

**Table 3:** Results of the generalized linear model analysis of the effect of selection treatment, experimental condition and their interaction on the log transformed juvenile cephalon length data

Cephalon length	Estimate	Std. Error	*T*‐value	*p*‐value
Intercept	6.019	0.012	495.339	**<0.001**
“Ambient 10 PSU” treatment	0.027	0.015	1.744	0.081
“Selected 16 PSU” treatment	−0.121	0.031	−3.903	**<0.001**
“High stress” experimental condition	0.045	0.019	2.41	**0.016**
“Low stress” experimental condition	0.002	0.019	0.112	0.911
“Ambient 10 PSU” treatment: “High stress” exp. condition	−0.146	0.026	−5.564	**<0.001**
“Selected 16 PSU” treatment: “High stress” exp. condition	NA	NA	NA	NA
“Ambient 10 PSU” treatment: “Low stress” exp. condition	−0.068	0.025	−2.739	**0.006**
“Selected 16 PSU” treatment: “Low stress” exp. condition	0.018	0.039	0.456	0.648

*T*‐value denotes the test statistic.

Significant values are highlighted in bold

### 3.4 Heritability

Heritability (*h* ^2^) estimated from offspring–midparent regression in “control” experimental condition of the “selected 4 PSU” treatment was 0.32 (±0.27) for juvenile growth and 0.18 (±0.14) for survival; heritability of the “selected 16 PSU” treatment was ‐ 0.02 (±0.62) for growth and 0.02 (±0.05) for survival (Supporting information Figure S1). Estimated heritability for juveniles hatched in “low stress” experimental condition from “selected 4 PSU” treatment was 1.36 (±0.61) for growth and 1.3 (±0.16) for survival (Supporting information Figure S2). Heritability for juveniles from the “selected 16 PSU” treatment in “low stress” experimental condition was ‐ 0.15 (±0.28) for growth and 0.13 (±0.11) for survival. In comparison, heritability in “high stress” experimental condition could only be calculated for juveniles hatched from the “selected 4 PSU” population and was ‐ 0.26 (±0.67) for growth and 0.49 (±0.43) for survival (Supporting information Figure S3). No juveniles hatched from the “selected 16 PSU” treatment in “high stress” experimental condition. In theory, heritability ranges between zero and one; however, due to random error estimates, it might deviate from this range (Conner & Hartl, 2004), as it was the case in our study.

## 4 DISCUSSION

As numerous species from the Ponto‐Caspian region originating from brackish environments established in brackish and freshwater habitats of the Baltic Sea and the Laurentian Great Lakes, in this study, we conducted selection experiments on a population of Ponto‐Caspian gammarid *Pontogammarus maeoticus* inhabiting 10 PSU in its native region to determine the adaptation capacity of this species to different salinities. Survival of adults, hatching success, juvenile survival, and heritability estimates indicated that selection to lower salinity than that of the population's ambient salinity is possible, although generation time in lower salinity conditions took slightly longer. On the contrary, selection to higher salinity was unsuccessful. However, interestingly even the population selected to higher salinity, “selected 16 PSU,” performed well in the “low stress” experimental condition. Due to the successful selection of the tested species to low salinity and freshwater conditions but not to high salinity, in addition to the geological history of the native region, we suggest that the *P. maeoticus* population tested, although currently inhabiting a brackish habitat, might be of freshwater origin. Consequently, if the majority of relict Ponto‐Caspian species are of freshwater origin, and therefore still retain alleles necessary for adaptation to freshwater conditions, the establishment of Ponto‐Caspian species in the Laurentian Great Lakes and other freshwater habitats should not be a surprise.

Heritability estimates obtained from offspring–midparent regression indicated possible selection of *P. maeoticus* to lower salinity than that of the ambient salinity of the population within few generations. Heritability of offspring from the “selected 4 PSU” treatment in “low stress” experimental condition was close to one, for both survival and growth, demonstrating that phenotypic variance was largely due to genetic causes (Conner & Hartl, 2004), and alluding on an existence of standing polymorphic variation for selection to act on. Hence, selection may be an effective factor increasing invasion success of this species, as the mean phenotype of the selected population might evolve rather rapidly, possibly allowing fast adaptation to low saline, and even freshwater habitats. Selection on standing genetic variation has already been suggested as one of the possible evolutionary scenarios for successful invasions, and was often observed in marine invertebrates (Briski et al., 2018; Rius, Turon, Bernardi, Volckaert, & Viard, 2015). Selection experiments on the copepod *E. affinis* also revealed an existing standing genetic variation for osmotic tolerance as basis for adaptation to fresh water (Lee, Remfert, & Chang, 2007). In contrast to selection to lower salinity, selection to higher salinity seems to be more difficult, if possible at all. The heritability estimates of the “selected 16 PSU” population, as well as of “selected 4 PSU” population, in “high stress” experimental condition indicated that phenotypic variation was mainly caused by environmental variance and nonadditive genetic variation (Conner & Hartl, 2004). Therefore, we suggest a possible lack of alleles for high salinity tolerance in the *P. maeoticus* population tested in this study. Selection to higher salinity might depend on new mutations and therefore would take much longer than selection on standing genetic variation (Barrett & Schluter, 2008).

In general, likely reason for the lack of genetic background for high salinity adaptation of species evolved in the Caspian Sea may be due to the geological history of the Ponto‐Caspian region, which underwent several changes in sea level and salinity (Dumont, 1998; Reid & Orlova, 2002). About 15 million years ago, the area of the Ponto‐Caspian basin was covered by a fully saline remnant of the Tethys Sea harboring mostly marine fauna of the “Mediterranean type” (Zenkevich, 1963). Ten million years ago, the Sarmatian Lake separated from the Tethyan Ocean and the salinity of the enclosed lake started to decrease, being several times almost completely dry during Glacial Maxima (i.e., from 2.5 million years ago to 10,000 years ago), and followed by freshwater flooding after ice melting at the end of each Glacial Maximum. During that period, several geological connections and disconnections of the region with the Mediterranean Sea occurred, causing additional salinity changes. Nowadays, salinity of the system is ranging from freshwater to marine (i.e., 0–30 PSU; Reid & Orlova, 2002; Zenkevich, 1963). Several authors suggested that river mouths, lagoons, and estuaries acted as a refuge for freshwater Caspian fauna to survive the periods of increased salinity in the basin (Cristescu et al., 2003; Zenkevich, 1963). Consequently, the majority of relict Ponto‐Caspian species might be of freshwater origin that are for millions of years adapting to higher salinity, and slowly spreading from those refugia to higher salinity of the Ponto‐Caspian region.

In the Caspian Sea, *P. maeoticus* occurs in salinity ranging from 0 PSU to 18 PSU (Nazarhaghighi et al., 2013). However, the population selected to lower salinity in this study had still relatively high mortality in freshwater conditions, although we believe that further selection steps would result in possible adaptation to fresh water. Recent molecular analysis of mitochondrial DNA revealed a marked genetic divergence between Caspian and Black Sea populations of *P. maeoticus*, as well as other Ponto‐Caspian crustaceans, such as the invasive cladoceran *Cercopagis pengoi*, suggesting there are two distinct phylogenetic groups between the two basins, which may have been separated for 5–7 million years (Cristescu et al., 2003). Furthermore, salinity tolerance experiments on 22 populations of eight species originating from the Ponto‐Caspian, Northern European, and Great Lakes‐St. Lawrence River regions demonstrated that different populations of one species can differ significantly in their salinity tolerance, highlighting that adaptation of spatially separated populations of the same species need to be taken into account for ecological analyses (Paiva et al., 2018). Selection experiments on the copepod *E. affinis* populations from different salinities further support this notion (Lee & Bell, 1999; Lee et al., 2007). Thus, adaptation potential and heritability may also differ widely among populations of the same species (Conner & Hartl, 2004). Therefore, we suggest that populations of *P. maeoticus* originating from different salinities may need different length and strength of selection for successful adaptation to freshwater conditions. In contrast, due to unsuccessful selection of the *P. maeoticus* population to higher salinity in this study, and the fact that the majority of Ponto‐Caspian nonindigenous species did not succeed to spread to more saline habitats in their native region through millions of years, nor did they invade the Mediterranean Sea (Paavola, Olenin, & Leppäkoski, 2005; Shiganova, 2010), we suggest a lack of necessary genetic background of those species for adaptation to fully marine environments, which further points to a freshwater origin of relict Ponto‐Caspian species.

In the past decades, Ponto‐Caspian species have successfully invaded brackish and freshwater habitats of the North and Baltic Seas and the Laurentian Great Lakes in much higher numbers than expected based on shipping frequency and environmental conditions among these regions, leading to the hypotheses that Ponto‐Caspian species may have inherent advantages over other species in colonizing new habitats, or that they are of freshwater origin (Bij De Vaate, Jazdzewski, Ketelaars, Gollasch, & Van Der Velde, 2002; Casties et al., 2016; Paiva et al., 2018; Ricciardi & Macisaac, 2000). Taking into account the results from our study and the above‐mentioned geological history of the Ponto‐Caspian region, we suggest that the majority of the Caspian relict fauna might be of freshwater origin. However, future selection studies using more species and populations, as well as molecular studies using state‐of‐the‐art techniques, such as high‐throughput sequencing, particularly for functional genes responding to salinity conditions should be conducted to confirm this hypothesis on a broader scale. Finally, as the majority of shipping ports (i.e., areas of highest introduction of nonindigenous species in aquatic habitats; Keller, Drake, Drew, & Lodge, 2011; Molnar, Gamboa, Revenga, & Spalding, 2008; Ricciardi, 2006) are located on river mouths and estuaries characterized by broad temporal salinity changes and frequently including freshwater conditions (Keller et al., 2011), we warn that future management strategies should take into account the adaptation capacity of Ponto‐Caspian species to different salinities, particularly to freshwater conditions.

## COMPETING INTERESTS

We declare no competing interests.

## AUTHOR CONTRIBUTIONS

E.B. conceived the study, E.B., N‐C.P. and F.P. designed the experimental set‐up, and N‐C.P. carried out experimental work and statistical analyses with the help of F.P. All authors were involved in writing the manuscript and gave final approval for publication.

## DATA ACCESSIBILITY

The full data set is available at PANGAEA, https://doi.org/10.1594/pangaea.887340. Adult survival data available at PANGAEA, https://doi.org/10.1594/pangaea.887336. Juvenile growth and survival data available at PANGAEA, https://doi.org/10.1594/pangaea.887339.
