# Corrigendum

**DOI:** 10.1002/ece3.5088

**Published:** 2019-04-16

In “Are Ponto‐Caspian species able to cross salinity barriers? A case study of the gammarid *Pontogammarus maeoticus,*” which was published in volume 8 issue 19, October 2018, the scales for panels a and b are missing in Figure 2. The correct image and caption are printed below.

**Figure 2:**
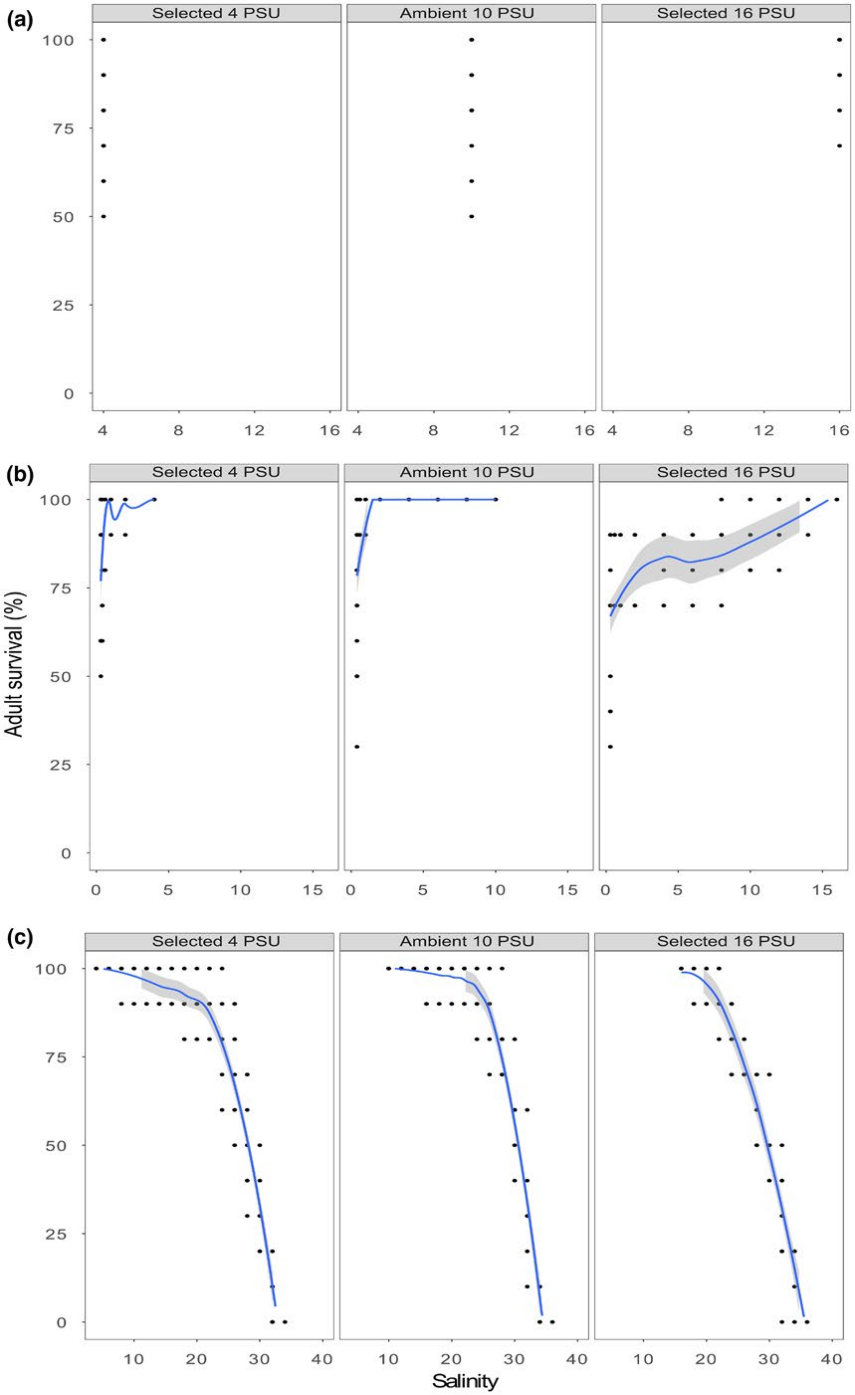
Survival of adults in “control” (a), “low stress” (b), and “high stress” (c) experimental conditions. Columns represent selection treatments (i.e., “selected 4 PSU,” “ambient 10 PSU,” and “selected 16 PSU,” respectively). Curves and respective confidence intervals (95%; gray area) were fitted using the method “loess” in R. Note the difference in scales for each panel
