# Supplementary material for: Are Ponto‐Caspian species able to cross salinity barriers? A case study of the gammarid Pontogammarus maeoticus
Source: Ecol Evol. 2018 Sep 5;8(19):9817–26. doi: 10.1002/ece3.4461 (PMC6202737; doi:10.1002/ece3.4461)
Supplement: Supplementary file 1 [file ECE3-8-9817-s001.doc]

**Are Ponto-Caspian species able to cross salinity barriers? – A case study of the gammarid *Pontogammarus maeoticus***

**SUPPLEMENTARY MATERIAL**

Nora-Charlotte Pauli^1,2,3^, Filipa Paiva^1,4^, Elizabeta Briski^1,*^

^1^GEOMAR Helmholtz Centre for Ocean Research Kiel, Düsternbrooker Weg 20, 24105 Kiel, Germany

^2^Christian-Albrechts Universität Kiel, Am Botanischen Garten 5-9, 24118 Kiel, Germany

^3^ present adress: Institute for Chemistry and Biology of the Marine Environment, Carl-von-Ossietzky University, Carl-von-Ossietzky-Straße 9-11, 26111 Oldenburg, Germany

^4^MARE – Marine and Environmental Sciences Centre, Quinta do Lorde Marina, Sítio da Piedade, 9200-044, Caniçal, Madeira Island, Portugal

*Corresponding author: Elizabeta Briski, Phone: +49 431 6001589, Fax +49 431 600 4402, e-mail: ebriski@geomar.de

**Table S1**. Results of pairwise comparisons of selection treatments and experimental condition of adult survival applying Dunn’s test with Bonferroni adjustment.

**Table S2**. Pairwise comparisons of selection treatment and experimental condition combinations of juvenile survival using the ‘lsmeans’ function in R applying a Bonferroni adjustment. SE and df denote standard error and degrees of freedom, respectively.

**Table S3**. Pairwise comparisons of selection treatment and experimental condition combinations of juvenile cephalon length using the ‘lsmeans’ function in R applying a Bonferroni adjustment. SE and df denote standard error and degrees of freedom, respectively.

**Figure S1**. Offspring-midparent regression for the “selected 4 PSU” (left) and “selected 16 PSU” (right) selection treatments in “control” experimental condition. The linear regression functions, R², p-value, and slope are shown. The slope of the regression was used as the heritability estimate (*h*²).

**Figure S2**. Offspring-midparent regression for the “selected 4 PSU” (left) and “selected 16 PSU” (right) selection treatments in “low stress” experimental condition. The linear regression functions, R², p-value and slope are shown. The slope of the regression was used as the heritability estimate (*h*²).

**Figure S3**. Offspring-midparent regression for the “selected 4 PSU” selection treatment in “high stress” experimental condition. The linear regression functions, R², p-value, and slope are shown. The slope of the regression was used as the heritability estimate (*h*²).

Table S1. Results of pairwise comparisons of selection treatments and experimental condition of adult survival applying Dunn’s test with Bonferroni adjustment.

| Contrast | z-value | p-value adj. |
| --- | --- | --- |
| “Selected 16 PSU” treatment - “Ambient 10 PSU” treatment | 3.511 | **< 0.001** |
| “Selected 4 PSU” treatment – “Ambient 10 PSU” treatment | 4.433 | **< 0.001** |
| “Selected 4 PSU” treatment – “Selected 16 PSU” treatment | 0.845 | 0.598 |
| “High stress” exp. condition – “Control” exp. condition | 3.096 | **0.003** |
| “Low stress” exp. condition – “Control” exp. condition | 0.248 | 1 |
| “Low stress” exp. condition – “High stress” exp. condition | - 2.590 | **0.014** |

Table S2. Pairwise comparisons of selection treatment and experimental condition combinations of juvenile survival using the ‘lsmeans’ function in R applying a Bonferroni adjustment. SE and df denote standard error and degrees of freedom, respectively.

| Treatment | Contrast | Estimate | SE | df | z-ratio | p-value |
| --- | --- | --- | --- | --- | --- | --- |
| *Experimental condition* | *Selection treatment* |  |  |  |  |  |
| “Control” | “Selected 4 PSU” - “Ambient 10 PSU” | 0.026 | 0.027 | NA | 0.978 | 0.985 |
| “Control” | “Selected 4 PSU” – “Selected 16 PSU” | - 0.171 | 0.072 | NA | - 2.388 | 0.051 |
| “Control” | “Ambient 10 PSU” – “Selected 16 PSU” | - 0.197 | 0.069 | NA | - 2.825 | **0.014** |
| “High stress” | “Selected 4 PSU” – “Ambient 10 PSU” | - 0.01 | 0.037 | NA | - 0.265 | 1 |
| “High stress” | “Selected 4 PSU” – “Selected 16 PSU” | - 0.169 | 0.297 | NA | - 0.57 | 1 |
| “High stress” | “Ambient 10 PSU” – “Selected 16 PSU” | - 0.159 | 0.297 | NA | - 0.536 | 1 |
| “Low stress” | “Selected 4 PSU”- “Ambient 10 PSU” | - 0.038 | 0.029 | NA | - 1.277 | 0.605 |
| “Low stress” | “Selected 4 PSU” – “Selected 16 PSU” | - 0.031 | 0.032 | NA | - 0.962 | 1 |
| “Low stress” | “Ambient 10 PSU” – “Selected 16 PSU” | 0.007 | 0.032 | NA | 0.222 | 1 |
| *Selection treatment* | *Experimental condition* |  |  |  |  |  |
| “Selected 4 PSU” | “Control” – “High stress” | 0.025 | 0.033 | NA | 0.77 | 1 |
| “Selected 4 PSU” | “Control” – “Low stress” | 0.065 | 0.03 | A | 2.122 | 0.102 |
| “Selected 4 PSU” | “High stress” – “Low stress” | 0.039 | 0.032 | NA | 1.239 | 0.646 |
| “Ambient 10 PSU” | “Control” – “High stress” | - 0.011 | 0.032 | NA | - 0.34 | 1 |
| “Ambient 10 PSU” | “Control” – “Low stress” | 0.001 | 0.026 | NA | 0.029 | 1 |
| “Ambient 10 PSU” | “High stress” – “Low stress” | 0.012 | 0.035 | NA | 0.33 | 1 |
| “Selected 16 PSU” | “Control” – “High stress” | 0.027 | 0.303 | NA | 0.09 | 1 |
| “Selected 16 PSU” | “Control” – “Low stress” | 0.205 | 0.072 | NA | 2.836 | **0.014** |
| “Selected 16 PSU” | “High stress” – “Low stress” | 0.178 | 0.297 | NA | 0.599 | 1 |

Table S3. Pairwise comparisons of selection treatment and experimental condition combinations of juvenile cephalon length using the ‘lsmeans’ function in R applying a Bonferroni adjustment. SE and df denote standard error and degrees of freedom, respectively.

| Treatment level | Contrast | Estimate | SE | df | z-ratio | p-value |
| --- | --- | --- | --- | --- | --- | --- |
| *Experimental condition* | *Selection treatment.* |  |  |  |  |  |
| “Control” | “Selected 4 PSU” – “Ambient 10 PSU” | - 0.027 | 0.015 | NA | - 1.744 | 0.244 |
| “Control” | “Selected 4 PSU” – “Selected 16 PSU” | 0.121 | 0.031 | NA | 3.903 | **< 0.001** |
| “Control” | “Ambient 10 PSU” – “Selected 16 PSU” | 0.148 | 0.03 | NA | 4.923 | **< 0.001** |
| “High stress” | “Selected 4 PSU” – “Ambient 10 PSU” | 0.119 | 0.021 | NA | 5.591 | **< 0.001** |
| “High stress” | “Selected 4 PSU” – “Selected 16 PSU” | nonEst | NA | NA | NA | NA |
| “High stress” | “Ambient 10 PSU” – “Selected 16 PSU” | nonEst | NA | NA | NA | NA |
| “Low stress” | “Selected 4 PSU” – “Ambient 10 PSU” | 0.042 | 0.019 | NA | 2.114 | 0.104 |
| “Low stress” | “Selected 4 PSU” – “Selected 16 PSU” | 0.104 | 0.023 | NA | 4.53 | **< 0.001** |
| “Low stress” | “Ambient 10 PSU” – “Selected 16 PSU” | 0.062 | 0.022 | NA | 2.847 | **0.013** |
| *Selection treatment* | *Experimental condition* |  |  |  |  |  |
| “Selected 4 PSU” | “Control” – “High stress” | - 0.045 | 0.019 | NA | - 2.41 | **0.048** |
| “Selected 4 PSU” | “Control” – “Low stress” | - 0.002 | 0.019 | NA | - 0.112 | 1 |
| “Selected 4 PSU” | “High stress” – “Low stress” | 0.043 | 0.021 | NA | 2.088 | 0.11 |
| “Ambient 10 PSU” | “Control” – “High stress” | 0.101 | 0.018 | NA | 5.499 | **< 0.001** |
| “Ambient 10 PSU” | “Control” – “Low stress” | 0.067 | 0.016 | NA | 4.151 | **< 0.001** |
| “Ambient 10 PSU” | “High stress” – “Low stress” | - 0.035 | 0.021 | NA | - 1.686 | 0.275 |
| “Selected 16 PSU” | “Control” – “High stress” | nonEst | NA | NA | NA | NA |
| “Selected 16 PSU” | “Control” – “Low stress” | - 0.019 | 0.033 | NA | - 0.591 | 0.555 |
| “Selected 16 PSU” | “High stress” – “Low stress” | nonEst | NA | NA | NA | NA |

**
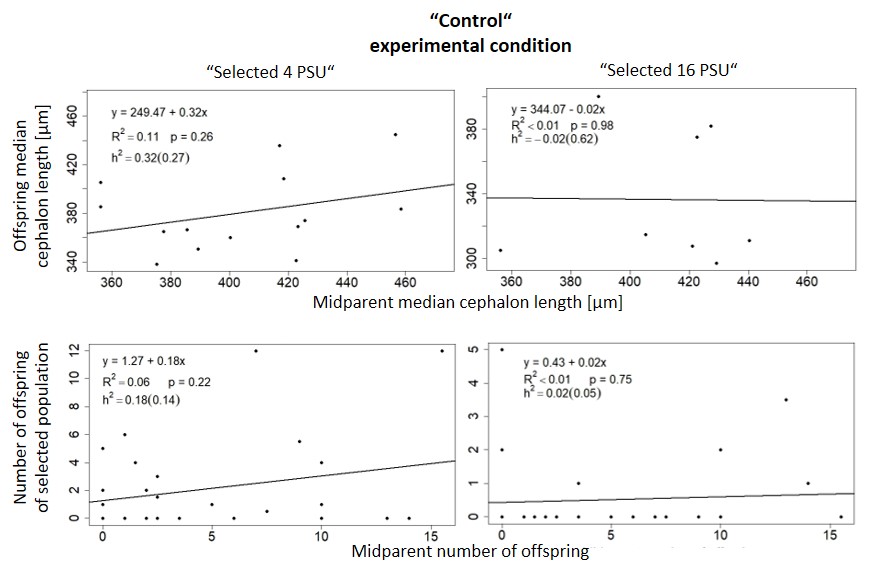
**

Figure S1. Offspring-midparent regression for the “selected 4 PSU” (left) and “selected 16 PSU” (right) selection treatments in “control” experimental condition. The linear regression functions, R², p-value, and slope are shown. The slope of the regression was used as the heritability estimate (*h*²).

**
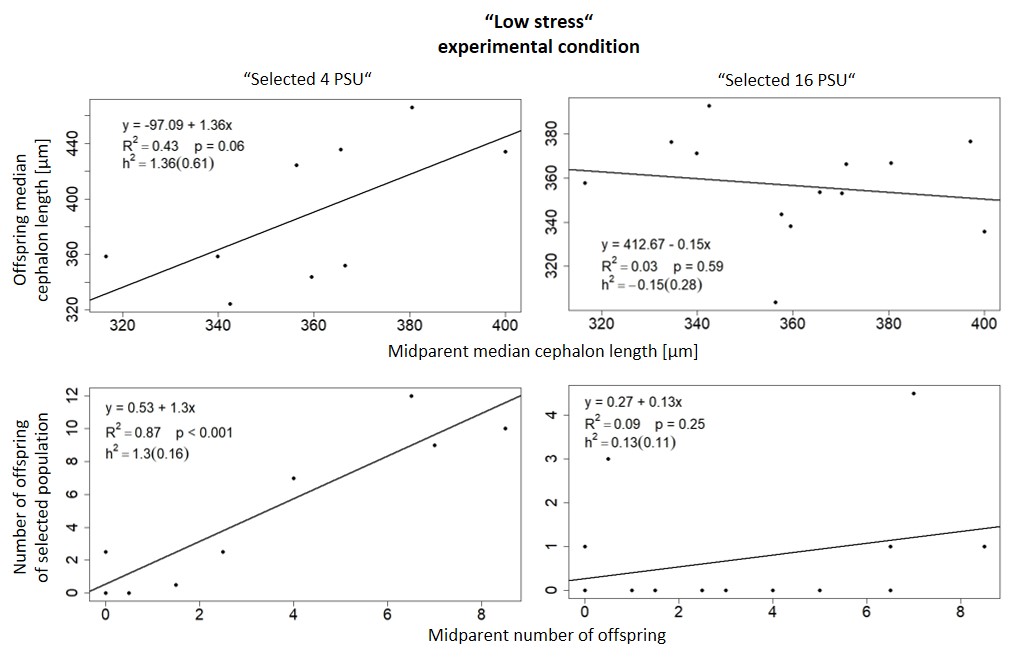
**

Figure S2. Offspring-midparent regression for the “selected 4 PSU” (left) and “selected 16 PSU” (right) selection treatments in “low stress” experimental condition. The linear regression functions, R², p-value and slope are shown. The slope of the regression was used as the heritability estimate (*h*²).

**
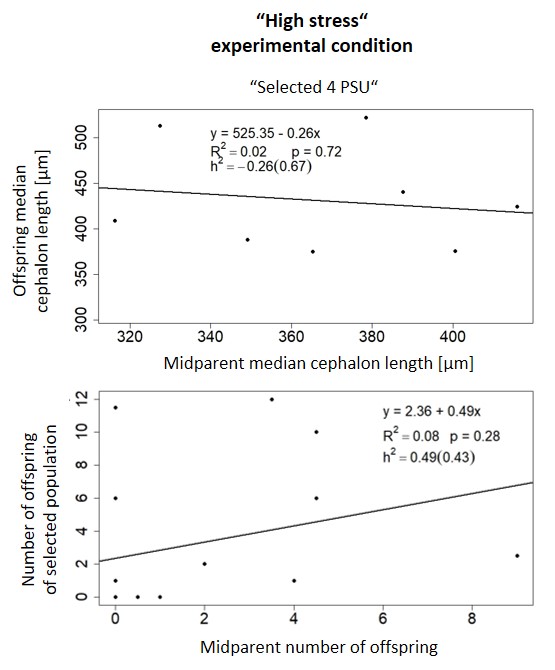
**

Figure S3. Offspring-midparent regression for the “selected 4 PSU” selection treatment in “high stress” experimental condition. The linear regression functions, R², p-value, and slope are shown. The slope of the regression was used as the heritability estimate (*h*²).
